# METTL3 affects the biological function of lung adenocarcinoma through the FGF2/PI3K/AKT /mTOR pathway

**DOI:** 10.3389/fonc.2024.1474701

**Published:** 2024-10-21

**Authors:** Shaoting Chen, Xiuqing Shen, Pengju Cao, Qianshun Chen, Rongxin Zhong, Yingping Cao

**Affiliations:** ^1^ Department of Clinical Laboratory, Fujian Medical University Union Hospital, Fuzhou, China; ^2^ Department of Clinical Laboratory, Fujian Provincial Hospital, Fuzhou, China

**Keywords:** LUAD, METTL3, m6A, FGF2, PI3K/AKT

## Abstract

**Introduction:**

This study aims to investigate the role of the m6A regulatory factor METTL3 in LUAD.

**Methods:**

By examining the expression of METTL3 in LUAD and conducting cellular functional experiments, the biological functions of METTL3 were discussed. mRNA-seq and MeRIP-qPCR were used to identify downstream target genes and pathways.

**Results:**

The expression level of METTL3 in LUAD is lower than that in the control group. The downregulation of METTL3 promoted the proliferation, migration, and invasion of LUAD cells, while overexpression of METTL3 results in the opposite effects. Furthermore, we found that FGF2 was negatively regulated by METTL3. Inhibiting FGF2 reversed the tumor-promoting effects caused by METTL3 downregulation in LUAD cells. Silencing METTL3 enhanced the stability of FGF2 mRNA. Silencing FGF2 resulted in reduced activity of the PI3K/AKT/mTOR signaling pathway in METTL3 knockdown LUAD cells.

**Discussion:**

In summary, our findings unveil an intricate signaling network involving METTL3/FGF2/PI3K/AKT/mTOR in LUAD and provide valuable insights into the molecular mechanisms underlying tumor progression, thus holding significant implications for targeted therapy and advancing LUAD research.

## 1 Introduction

Lung Adenocarcinoma (LUAD) is the main type of non-small cell lung cancer. Despite the new markers and treatments, LUAD morbidity and mortality are commonly observed (1, 2). N6-methyladenosine (m6A) is one of the most commonly observed RNA modifications (3) and is mainly composed of proteins, such as m6A methyltransferases, m6A demethylases, and m6A-binding proteins, which function via reversible regulation (4). m6A modifications are written by m6A methyltransferase, erased by m6A demethylase, and read by m6A binding proteins (5). Dysregulation of m6A is commonly observed in various cancer types (6) and plays an important role in cancer initiation, progression, metastasis, metabolism, drug resistance, immune evasion (7) and Cancer stem cells (CSCs) self-renewal (8). Methyltransferase is a multi-component complex composed of Methyltransferase like 3 (METTL3), methyltransferase like 14 (METTL14) and RNA Binding Motif Protein 15B (WTAP), of which METTL3 plays a major catalytic role (9, 10). Related to tumor heterogeneity or the different model systems used in the previous studies, METTL3 has oncogenic or tumor suppressor functions in different groups (5). METTL3 plays an essential role in cancer mainly by affecting RNA splicing, export, localization, translation, stability (6, 10–12).

Currently, the role of METTL3 in tumor progression remains contentious, with controversies potentially stemming from variations in cell types, modeling techniques, disease states, or other potential regulatory factors. Therefore, conducting a larger number of clinical tissue sample studies can provide a more comprehensive understanding of the specific involvement of METTL3 in LUAD, which holds significant implications for elucidating its mechanistic actions in this particular cancer type.

The pathological formation of new blood vessels, known as angiogenesis, is a characteristic feature observed in cancer as well as various ischemic and inflammatory diseases (13). The underlying mechanism by which m6A modified enzymes influence angiogenesis in cancer is gradually being elucidated. Increasing evidence suggests a significant impact of m6A on tumor angiogenesis, with a reduction in m6A levels leading to vascular remodeling and progression of metastasis (14). Fibroblast growth factor 2 (FGF2) is a potent angiogenic factor, promoting the formation of new blood vessels. In certain situations, FGF2 activates inflammatory cascades, leading to the abnormally high expression of vascular endothelial growth factor 1(VEGF1) and FGF2 in lung tissue structures. This abnormal expression is a major driving factor for abnormal angiogenesis and the development of lung cancer (15). However, the association between METTL3 and FGF2 remains to be explored.

## 2 Materials and methods

### 2.1 Patients specimens

Cancer tissues (n=92) and their corresponding cancer-adjacent tissues were collected from patients at Fujian Provincial Hospital (Fuzhou, China). Patients with LUAD were diagnosed histopathologically without chemotherapy or radiotherapy. This study was approved by the Research Ethics Committee of Fujian Provincial Hospital (K-2021-040-04) and conform to the ethical standards of the 1964 Declaration of Helsinki.

### 2.2 Cell culture and transfection

Human LUAD cell lines A549 and PC9 and normal human lung epithelial cell line BEAS-2B were obtained from the American Type Culture Collection. Both of them were cultured in PRIM-1640 medium (Hyclone, USA) with 10% fetal bovine serum (Gibco, USA) and incubated in a 37°C atmosphere with 5% CO_2_. A549 cells were seeded in 6-well plates at a density of 5 × 10^4^ cells/well and cultured for 24 h. METTL3 control, METTL3 inhibitor, METTL3 mimics, and 0.5 µg/mL polybrene (Genepharma, China), were mixed in PRIM-1640 medium according to the manufacturer’s instruction. This mixture was used to infect A549 cells. A total of 4 groups were transfected, including blank group, unloaded virus group(Control), METTL3 knockdown group (METTL3-KD)and METTL3 overexpression group(METTL3-OE). siFGF2 (Genepharma, China) was used to infect A549 METTL3-KD cells. Three groups were infected including Control-siNC, METTL3_KD+siNC (transient siNC plasmid transfer in METTTL3-KD stable cell line), and METTL3_KD+siFGF2 (transient FGF2 plasmid silencing in METTTL3-KD stable cell line).

### 2.3 RNA extraction, reverse transcription, and quantitative polymerase chain reaction

RNA was extracted using TRIzol Reagent (Invitrogen, USA). A PrimeScript RT Reagent Kit (TaKaRa, Japan) was used for reverse transcription. TB Green® Premix Ex Taq™ II (TaKaRa, Japan) was used for qPCR. Experimental manipulations and reaction temperature conditions were performed according to the manufacturer’s instructions. GAPDH was used as the internal standard. The primers are following (SunYa, China): METTL3-Forward: CTGCAACGCATCATTCGGAC, METTL3-Reverse: AGACCCTGGTTGAAGCCTTG; GAPDH-Forward: GGTGTGAACCATGAGAAGTATGA, GAPDH-Reverse: GAGTCCTTCCA CGATACCAAAG.

### 2.4 Methylated RNA immunoprecipitation-qPCR

MeRIP experiments were performed using the EpiQuik CUT&RUN m6A RNA Enrichment Kit (Epigentek, New York), according to the manufacturer’s protocols. RNA was calculated using RT-qPCR description like 2.3. The primers are following (SunYa, China): FGF2-Forward: GCACTGAAACGAACTGGGCA, FGF2-Reverse: GAAATGAGATTAGATGT GGC.

### 2.5 Cell counting kit-8

CCK8 was used to test cell viability (Beyotime, China). Three thousand cells per well were plated in 96-well plates and incubated for 0, 24, 48, and 72 h. The old medium was discarded, and a 10% solution of CCK8 was added. The plates were incubated at 37°C for an additional hour. Absorbance was measured at 450 nm using a Multiskan spectrophotometer. During detection, the liquid was shaken, and the bubbles were removed.

### 2.6 Wound-healing assay

About 1×10^6^ cells were seeded to each well of a 6-well plate. Cells were scraped using a sterile pipette tip once they reached over 95% confluency for the wound-healing assay. The detached cells were washed with PBS and incubated at 37°C for 48 h. Photographs were taken at the start (0 h) and after 48 h to determine the percentage of wound closure. The scratch area was calculated using ImageJ software.

### 2.7 *In vitro* invasion assays

Approximately 5×10^4^ cells were seeded in the upper chamber of a polycarbonate-coated trans well filter coated with Matrigel. The cells were then cultured at 37°C for 48 h. Next, the medium in the upper and lower chambers was discarded, and 4% paraformaldehyde solution was added to the lower chamber to fix the cells for 30 min. Cells that did not pass through the membrane were removed using a cotton swab. A 1% crystal violet solution was added to stain the cells, and the mixture was incubated for 15 min. Images were captured under a microscope, and the average value of five random ×400 fields per well was calculated. This process was repeated five times for each group. The results are presented as the mean ± standard deviation.

### 2.8 Online database analysis

Gene Expression Profiling Interactive Analysis (GEPIA; http://gepia.cancer-pku.cn) is a web server for analyzing RNA sequencing expression data from The Cancer Genome Atlas Program (TCGA) and GTEx projects. Using GEPIA, we explored the expression of METTL3 in LUAD. A Kaplan–Meier plotter (KM, http://kmplot.com/analysis) was used to assess the correlation between METTL3 and tumor survival (16). SRAMP (http://www.cuilab.cn/) is used to prediction FGF2 methylation modification sites (17).

### 2.9 Western blot

Cancerous tissue, para-cancerous tissue, and cells were harvested and lysed with RIPA buffer supplemented with protease and phosphatase inhibitors on ice for 30 min. Protein quantities were determined using enhanced BCA protein assay kit (Beyotime Biotechnology), ensuring consistent loading (20ug total). Equal amounts of protein were electrophoretically separated on 10% polyacrylamide gels before being transferred to polyvinylidene fluoride membranes. The membranes underwent with 5% skim milk for blocking. Antibodies targeting METTL3, p‐PI3K, PI3K, p‐AKT, AKT, mTOR, p-mTOR and GAPDH (Cell Signaling Technology; diluted 1:1000) were incubated overnight at 4°C. Subsequently, the membranes were washed three times, followed by incubation with HRP-conjugated affinipure goat anti-rabbit IgG (H + L) (1:10000, SA00001-2, proteintech) secondary antibodies for 1.5 h at room temperature. The membranes underwent a triple wash using TBST. Visualization of protein bands was achieved using an ECL kit (ThermoFisher Scientific).

### 2.10 RNA sequencing and analysis

To examine the regulation of related factors in A549 cells by METTL3, we performed transcriptome sequencing of virus-transfected cells, completed by Shanghai Megi Biological. Data were analyzed using the online platform Majorbio Cloud Platform (www.majorbio.com).

### 2.11 mRNA stability

To detect the RNA stability in cells, actinomycinD (Act-D; 5 mg/mL; Sigma, USA) was administrated to cells. At the indicated time point, RNA was isolated and analyzed by RT-qPCR.

### 2.12 Statistical analysis

Figures were generated using GraphPad Prism version 6.02. An unpaired Student’s t-test or paired t-test was used for difference analysis. Spearman’s correlation analysis was used to describe the correlation between quantitative variables without a normal distribution. Significance was set at P < 0.05. Statistical analysis was performed using SPSS 22.0 and R 4.1.3.

## 3 Results

### 3.1 Downregulation of METTL3 expression in LUAD tissues and cells

**Figure 1:**
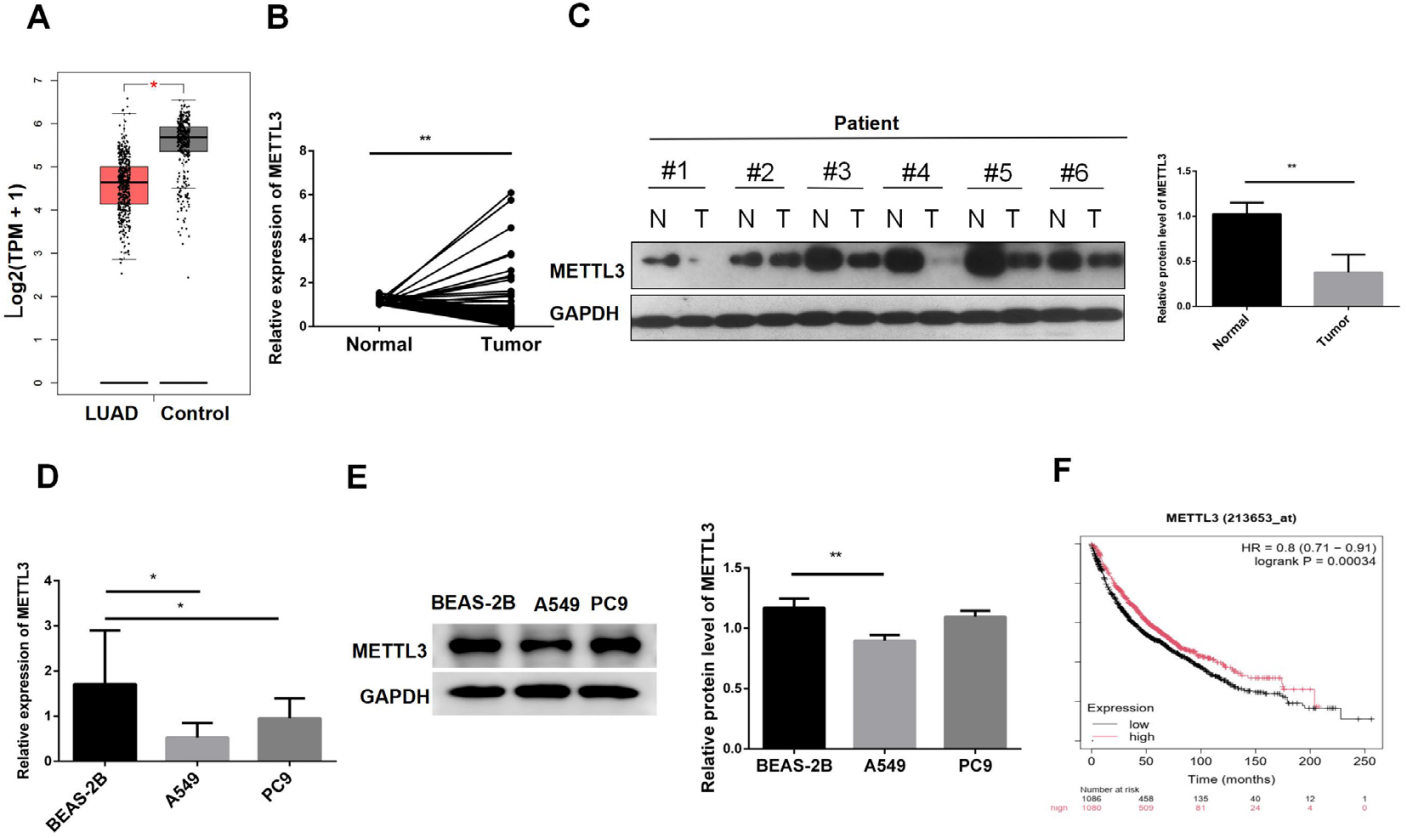
METTL3 is downregulated in lung adenocarcinoma tissues and cells. **(A)** GEPIA showed that METTL3 was decreased in LUAD. **(B)** METTL3 mRNA expression was decreased in LUAD patient tissues. **(C)** METTL3 protein expression was decreased in LUAD patients. **(D)** The METTL3 mRNA level in normal lung epithelial cells BEAS-2B was lower than that in LUAD cells A549 and PC9. **(E)** The METTL3 mRNA protein level in BEAS-2B was lower than that in A549; there was no difference with PC9. **(F)** Low expression of METTL3 was associated with poor prognosis in LUAD patients. *P < 0.05, **P < 0.01.

The GEPIA results showed that METTL3 was expressed at lower levels in tumor tissues (**Figure 1A**). Subsequently, we validated the expression levels of METTL3 mRNA in 92 pairs of tumor and adjacent normal tissues (**Figure 1B**). The results indicated that METTL3 was generally expressed at lower levels in tumor tissues. To verify METTL3 protein expression levels, we performed western blot analysis on 6 pairs of tissues. The results showed that METTL3 protein was expressed at lower levels in tumor tissues (**Figure 1C**). We further assessed METTL3 mRNA and protein expression at the cellular level. The results indicated that METTL3 mRNA was expressed at lower levels in cells A549 and PC9 compared to BEAS-2B (**Figure 1D**). Western blot results also indicated that METTL3 was downregulated in A549 (**Figure 1E**). Additionally, we analyzed the impact of METTL3 on the survival time of LUAD patients. The results suggested that lower METTL3 expression levels were associated with poorer prognosis (**Figure 1F**). In summary, these analyses indicate that METTL3 is downregulated in tumors and suggest a poor prognosis.

### 3.2 Biological functions of METTL3 in LUAD

**Figure 2:**
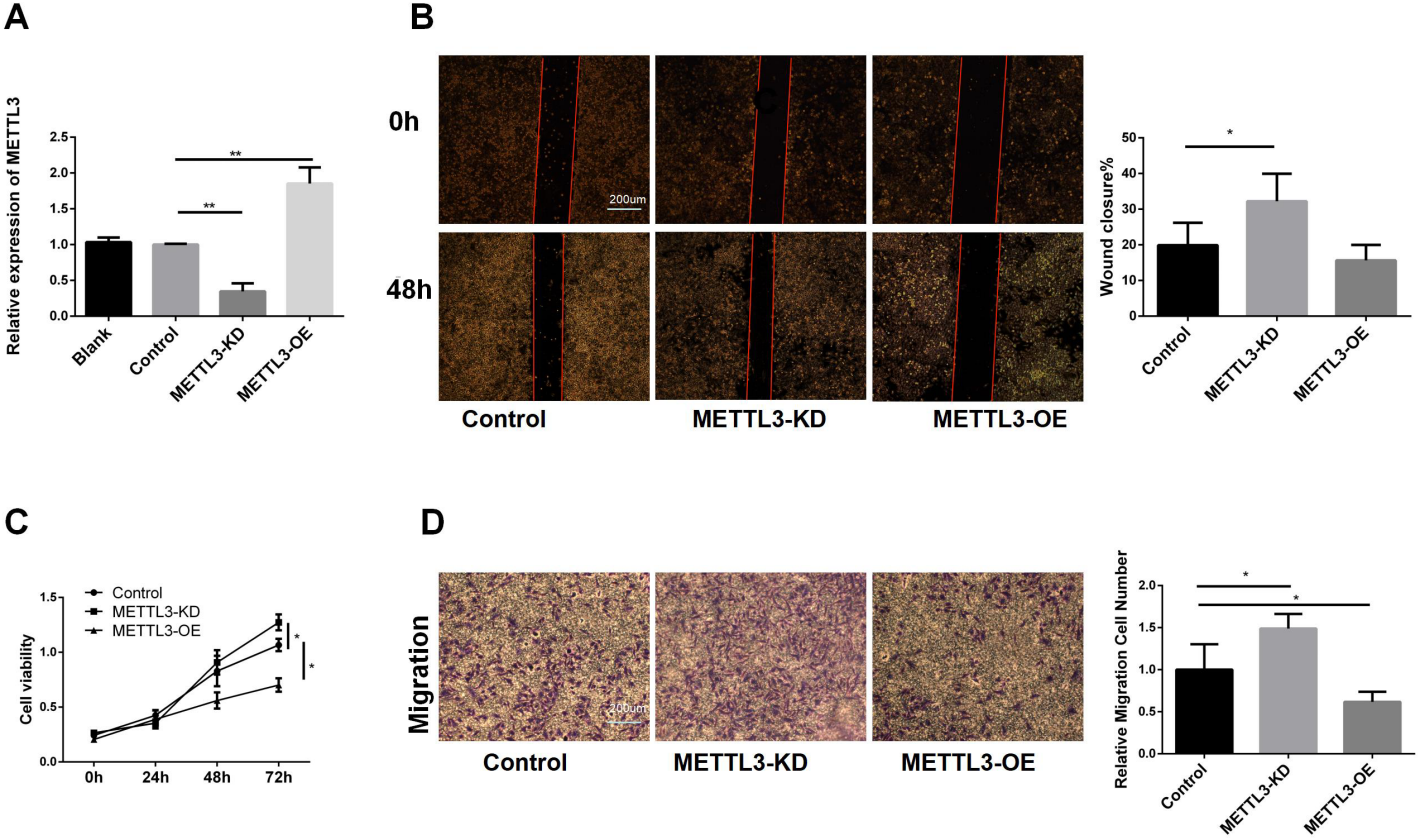
Biological Functions of METTL3 in LUAD. **(A)** RT-qPCR showed that the stable cell lines of METTL3 knockdown and overexpression were successfully constructed. **(B)** Compared with the control group, knockdown of METTL3 accelerated scratch healing, while overexpression of METTL3 had no difference. **(C)** Compared with the control group, knockdown of METTL3 promoted cell proliferation, while overexpression of METTL3 inhibited proliferation. **(D)** Compared with the control group, knockdown of METTL3 promoted cell migration, while overexpression of METTL3 inhibited migration. *P <0.05, **P <0.01.

To further validate the role of METTL3 in LUAD, we constructed A549 cell lines with METTL3 knockdown and overexpression. The RT-qPCR results (**Figure 2A**) of indicated that we have a successful infection. Subsequently, we conducted a cell scratch assay (**Figure 2B**). The scratch assay results showed that the scratch healing area decreased after METTL3 knockdown, suggesting that downregulation of METTL3 promotes cell migration. Using the CCK8 assay (**Figure 2C**), we observed that METTL3-KD increased tumor cell viability, while overexpression of METTL3 decreased tumor cell viability, indicating that METTL3 inhibits tumor cell proliferation. To explore the effect of METTL3 on tumor migration, we performed transwell assays. The results showed that the number group of METTL3-KD cells crossing the transwell subventricular membrane was greater more than that of the control group, and while the METTL3-OE group crossing was less than that of the control group (as shown in **Figure 2D**). Collectively, it indicated that knockdown of METTL3 promoted the proliferation and migration of A549 cells, and overexpression of METTL3 inhibited the proliferation and migration of A549 cells.

### 3.3 The result and analysis of RNA-seq

**Figure 3:**
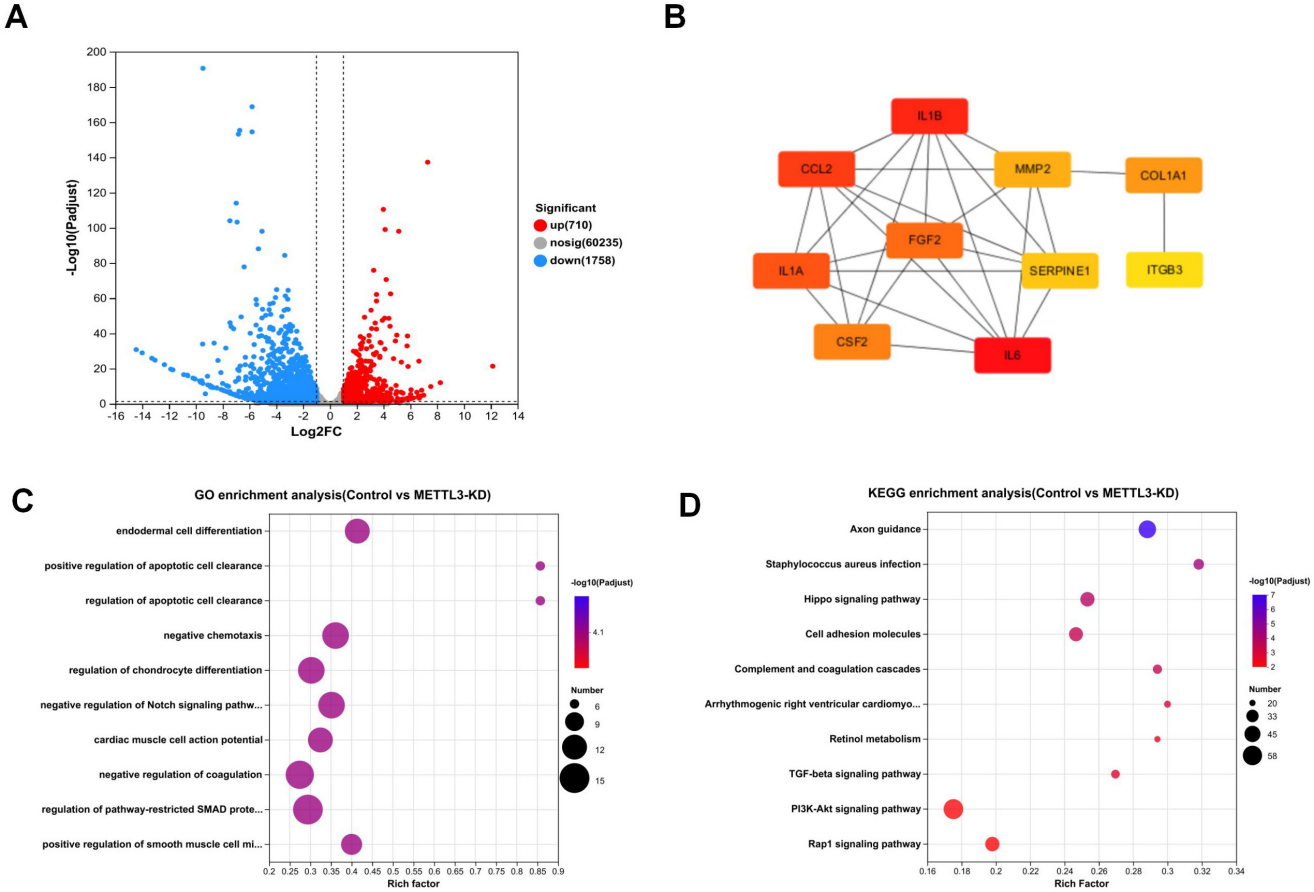
The result and analysis of RNA-seq. **(A)** Volcano plot shows that knockdown of METTL3 leads to changes in the expression of 2,468 genes. **(B)** Top 10 hub genes among differentially expressed genes. **(C)** GO enrichment analysis plot comparing Control vs. METTL3-KD. **(D)** KEGG enrichment analysis plot comparing Control vs. METTL3-KD.

As shown in **Figure 3A**, RNA-seq analysis revealed that compared to the control, METTL3 knockdown (METTL3-KD) resulted in changes in the expression of 2,468 genes, with 710 genes downregulated and 1,758 genes upregulated. In contrast, only 27 genes were differentially expressed in METTL3 overexpression (OE) compared to the control. Consequently, we chose METTL3-KD for further analysis. **Figure 3B** displays the top ten hub genes identified, all of which play significant roles in tumors. According to Gene Ontology (GO) analysis (**Figure 3C**), the differentially expressed genes were primarily enriched in processes such as endodermal cell differentiation, positive regulation of apoptotic cell clearance, and regulation of epithelial to mesenchymal transition. The Kyoto Encyclopedia of Genes and Genomes (KEGG) is a comprehensive knowledge base for systematically analyzing gene functions and linking genomic information with functional information. KEGG analysis (**Figure 3D**) revealed that the differentially expressed genes were associated with cancer-related pathways, including the PI3K/AKT signaling pathway and the TGF-β signaling pathway. These pathways are closely related to tumor cell growth and differentiation. Our findings suggest that downregulating METTL3 induces alterations in the cellular function and downstream pathways of A549 cells, which are significantly correlated with tumor progression. This implies that the dysregulation of METTL3 plays a pivotal role in A549.

### 3.4 METTL3 mediated the methylation modification of FGF2

**Figure 4:**
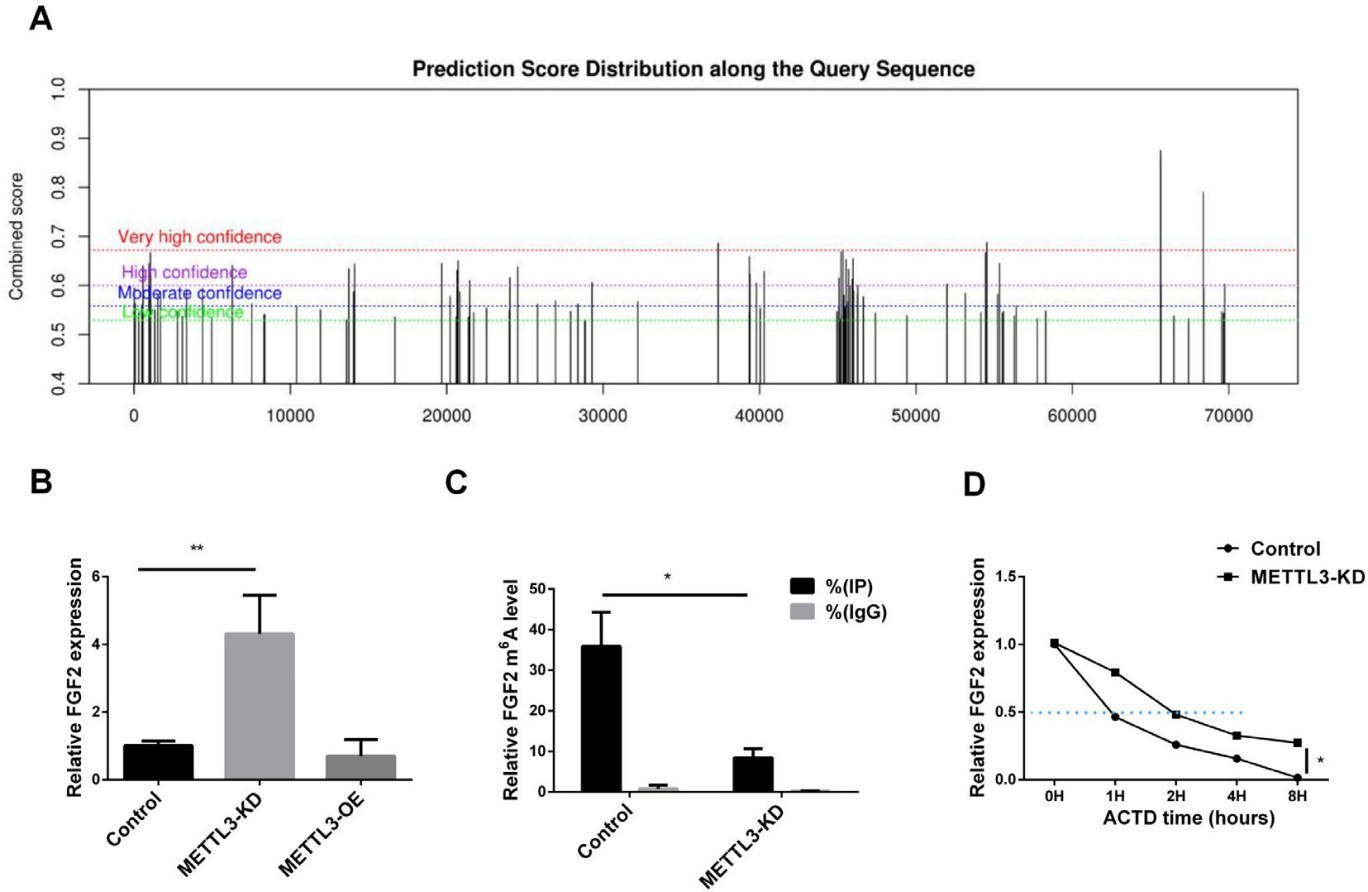
METTL3 mediated the methylation modification of FGF2. **(A)** FGF2 methylation site prediction shows that there are a large number of methylation sites in the FGF2 sequence. **(B)** RNA-seq results show that FGF2 mRNA expression is upregulated in A549 cells with METTL3 knockdown. **(C)** MeRIP-qPCR results show that FGF2 mRNA m6A levels are mediated by METTL3. **(D)** FGF2 mRNA half-life is prolonged in METTL3 knockdown cells. *P <0.05, **P <0.01.

To investigate whether the regulation of FGF2 by METTL3 is related to m6A modification, we predicted the m6A modification sites of FGF2. As shown in **Figure 4A**, there are abundant m6A modification sites on the FGF2 sequence. Subsequently, we confirmed the expression levels of FGF2 in METTL3-KD and METTL3-OE cell lines. As shown in **Figure 4B**, FGF2 expression was upregulated in METTL3-KD cell lines, indicating that silencing METTL3 induced an increase in FGF2. Using MeRIP-qPCR (**Figure 4C**), We found that the methylation level of FGF2 was significantly downregulated after METTL3 knockdown, suggesting that FGF2 is subject to METTL3-mediated methylation modification. To determine whether METTL3 affects the stability of FGF2 RNA, we conducted a half-life assay for FGF2. The results (**Figure 4D**) indicated that the half-life of FGF2 was prolonged in METTL3-KD cell lines, suggesting that METTL3 promotes the degradation of FGF2 through methylation modification.

### 3.5 Inhibition of FGF2 abrogates the effects of mettl3 knockdown on proliferation, migration and invasion of LUAD cells

**Figure 5:**
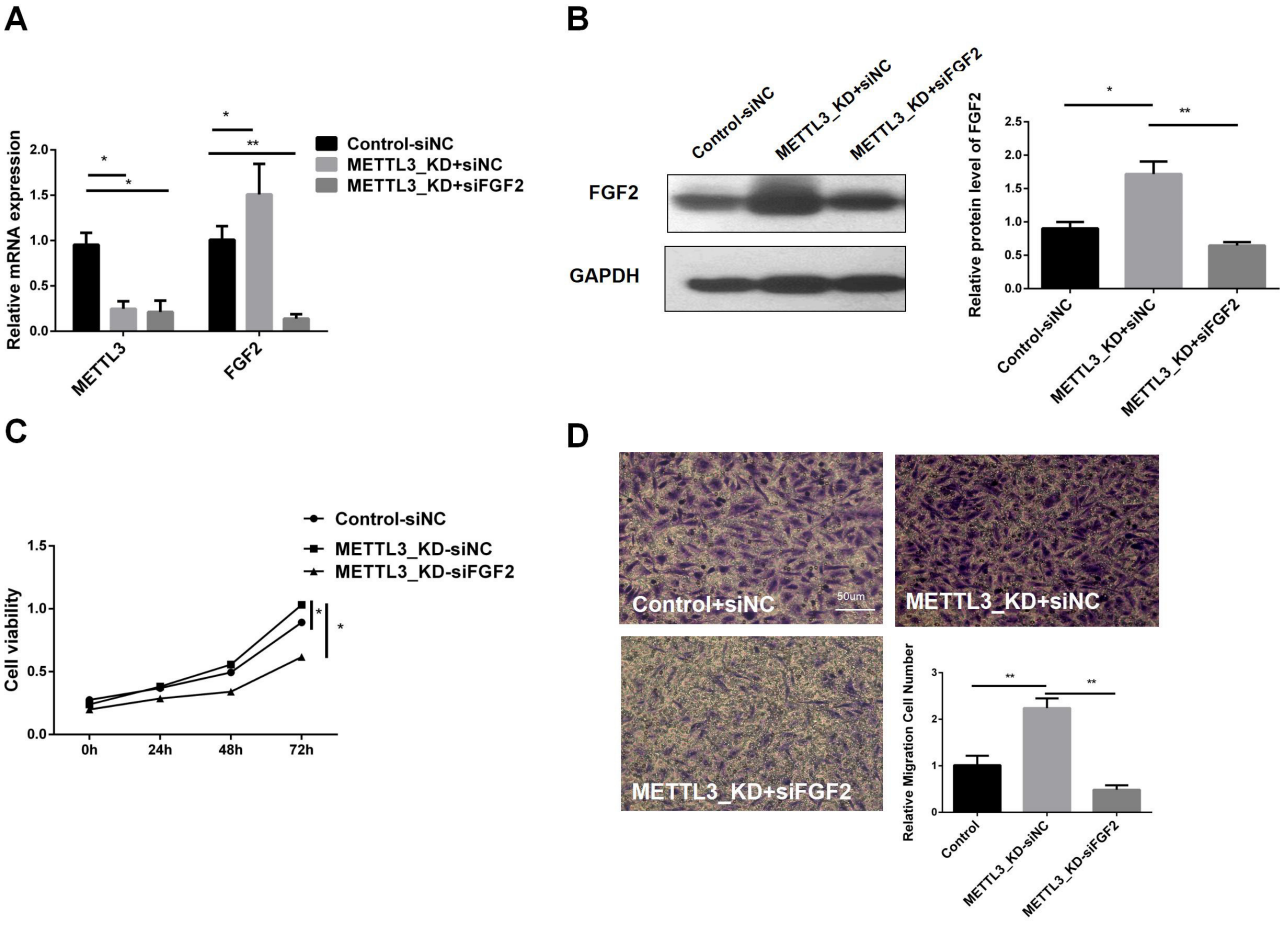
Inhibition of FGF2 Eliminates the Effects of METTL3 Knockdown on the Proliferation, Migration, and Invasion of NSCLC Cells. **(A)** RT-qPCR validation of FGF2 knockdown and control constructs in METTL3-KD. **(B)** Compared with the control-siNC group, FGF2 protein was highly expressed in METTL3 knockdown cell lines, but there was no significant difference in METTL3 knockdown combined with FGF2 silencing cell lines. **(C)** Silencing FGF2 resulted in decreased cell proliferation in METTL3-KD cells. **(D)** Silencing FGF2 resulted in decreased cell invasion in METTL3-KD cells. *P <0.05, **P <0.01.

To understand whether METTL3 plays a role in LUAD by regulating FGF2, we transfected siFGF2 into the METTL3-KD cell line. The infection efficiency was verified by RT-qPCR and western blot, and the results are shown in **Figures 5A, B**. In the constructed cell line, we performed CCK8 cell viability assay and transwell experiment. **Figure 5C** shows that the tumor cell viability in METTL3_KD+siFGF2 was significantly lower than that in METTL3_KD+siNC, indicating that silencing FGF2 can reduce the cell proliferation activity of METTL3-KD. **Figure 5D** shows that the migration ability in METTL3_KD+siFGF2 was lower than that in METTL3_KD+siNC, indicating that silencing FGF2 can reduce the cell migration ability of METTL3-KD. In short, FGF2 alleviated the malignant progression of tumors caused by METTL3 in LUAD cells. In addition, compared with Control+siNC, the cell viability and invasion ability in METTL3_KD+siNC were improved, further verifying our previous conclusions.

### 3.6 FGF2 participates in PI3K/AKT/mTOR pathway in LUAD

**Figure 6:**
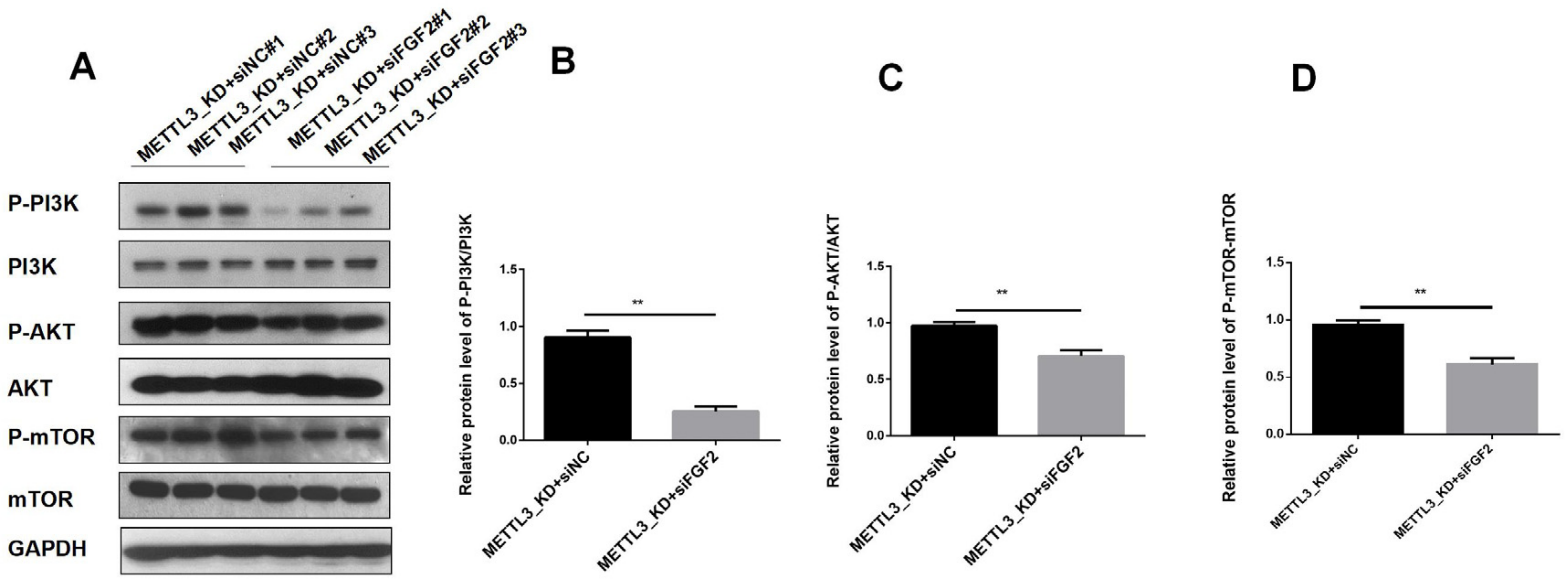
The result of expression of PI3K/AKT T/mTOR pathway in LUAD. **(A)** In the A549 cell line of METTL3-KD, the activity of the PI3K/AKT T/mTOR pathway decreased after FGF2 silencing. **(B)** The phosphorylation level of PI3K decreased after FGF2 silencing. **(C)** The phosphorylation level of AKT decreased after FGF2 silencing. **(D)** The phosphorylation level of mTOR decreased after FGF2 silencing **P<0.01.

To elucidate the downstream pathways impacted by METTL3-mediated FGF2 upregulation in LUAD cells, we suppressed FGF2 expression in METTL3 knockdown cell lines. Our RNA-seq analysis revealed that differentially expressed genes in LUAD following METTL3 knockdown were significantly enriched in the PI3K/AKT pathway. Thus, we sought to investigate whether METTL3/FGF2 influenced this pathway's activity level. The results (**Figure 6**) demonstrated that silencing led to inhibition of the PI3K/AKT/mTOR pathway activation level in METTL3 knockdown LUAD cells. In summary, our findings unveil a signaling network involving METTL3/FGF2/PI3K/AKT/mTOR in LUAD and shed light on the molecular mechanism underlying tumor malignant progression while holding great significance for targeted therapy in research on LUAD.

## 4 Discussion

m6A is a significant RNA modification strongly correlated with cancer and is subject to stringent regulation by m6A regulators (18). Dysregulation of m6A modification and its associated enzymes frequently occurs across various cancer types. METTL3 plays a complex dual role in cancer development. Its tumor-suppressive effects have been reported in lung cancer (19), colorectal cancer (20), and renal cell carcinoma (21) while its cancer-promoting effects have been reported in the above mentioned tumors (22–25). Wu et al. (19) found that METTL3 played a tumor suppressor role by enhancing the translation of the tumor suppressor gene FBXW7 mRNA. Currently, seeking treatment methods for tumors from the perspective of m6A has become a hot topic. For example, the combination of m6A modulators and chimeric antigen receptor T-cell (CAR-T) therapy (26), which has transformed the landscape of cancer treatment, is also gaining attention (27). The modulation of RNA methylation levels and the reversible m6A modification on transcripts may serve as novel epigenetic indicators with significant biological implications and potential therapeutic targets.

Our analysis conducted using the GEPIA database supported the significant downregulation of METTL3 expression in LUAD. The validity of this conclusion was confirmed through the assessment of mRNA and protein levels in lung adenocarcinoma tissues in vivo and cells in vitro. Establishing the expression of METTL3 in LUAD will contribute to a more comprehensive understanding of its functional role within this context. Furthermore, KM survival prognostic analysis revealed that higher METTL3 expression was associated with a poorer prognosis. Based on these results, we hypothesized that METTL3 plays a crucial role in the development and progression of LUAD. Moreover, the knockdown of METTL3 facilitated the invasion and migration of A549 cells. RNA-seq results revealed that knockdown of METTL3 induced significant alterations in multiple crucial biological processes and downstream pathways, particularly the PI3K/AKT signaling pathway and cancer-related pathways. These findings elucidate the association between decreased levels of METTL3 and an unfavorable prognosis, thereby confirming the conjecture regarding the pivotal role played by METTL3 in LUAD.Overexpression of FGF promoted angiogenesis (28). Inhibitors and drugs targeting FGF2 can effectively inhibit the development of lung cancer (29). The m6A modification of FGF2 is currently gaining attention. YTHDF3 controls FGF2 translation in an m6A-dependent manner, directly targeting FGF2 by affecting the stability of FGF2 protein, thus influencing the malignant progression of breast cancer cells (30). Our RNA-seq found that FGF2 is our HUB gene, which is highly expressed in METTL3-KD cell lines. This piqued our interest, prompting us to investigate the potential correlation between METTL3 knockdown and the pathogenesis of LUAD by examining its impact on FGF2 expression. Subsequently, we discovered that FGF2 mRNA has abundant m6A sites, and through MeRIP-qPCR, we confirmed that METLL3 can mediate mA modification of FGF2 mRNA. To further investigate whether METTL3 affects LUAD functions through FGF2, we silenced FGF2 in the METTL3-KD cell line. Functional assays showed that silencing FGF2 slowed down tumor growth and reduced invasion. Our findings have important implications for the development of targeted therapeutic options for LUAD.

The PI3K/AKT signaling pathway is a major pathway in various types of cancer, influencing cancer progression by controlling cell survival, angiogenesis, inflammation, metastasis, and metabolism (31). The PI3K/AKT pathway is involved in the proliferation and metastasis of LUAD (32). After METTL3 knockdown using RNA-seq, we observed a significantly enriched differential gene expression in the PI3K/AKT signaling pathway. Our objective was to investigate the impact. FGF2/PI3K/AKT pathway promotes polarization of M2 macrophages, thereby promoting corneal neovascularization (33). The FGF2/PI3K/AKT pathway may be involved in neurovascular repair in ischemic stroke (34). Our study found that the Silencing FGF2 leads to the inactivation of PI3K/AKT signaling pathway in METTL3 knockdown cells. This indicates that the reduction of METTL3 promotes LUAD development by upregulating FGF2 and activating the PI3K/AKT pathway. Our findings suggest that dysregulation of METTL3-mediated m6A modification may impact LUAD progression by promoting tumor angiogenesis. Suggestion is made to explore treatment options for LUAD through the modulation of m6A regulators.

## 5 Conclusion

In LUAD, the expression of METTL3 is reduced, indicating that METTL3 is a potential molecular biological diagnostic marker for LUAD. Knockdown of METTL3 promotes the proliferation and migration in LUAD cells. By silencing FGF2, the proliferation and migration induced by METTL3 downregulation are weakened, Silencing FGF2 leads to the inactivation of PI3K/AKT/mTOR signaling pathway in METTL3 knockdown cells. It is believed that the loss of METTL3 may promote the progression of LUAD by upregulating the FGF2/PI3K/AKT/mTOR axis. Our findings are of great significance for the development of targeted therapeutic approaches for LUAD.

## Data Availability

The raw data supporting the conclusions of this article will be made available by the authors, without undue reservation.
